# Solid-Phase Extraction Embedded Dialysis (SPEED), an Innovative Procedure for the Investigation of Microbial Specialized Metabolites

**DOI:** 10.3390/md19070371

**Published:** 2021-06-26

**Authors:** Phuong-Y. Mai, Géraldine Le Goff, Erwan Poupon, Philippe Lopes, Xavier Moppert, Bernard Costa, Mehdi A. Beniddir, Jamal Ouazzani

**Affiliations:** 1 CNRS, Institut de Chimie des Substances Naturelles, UPR 2301, 1, Avenue de la Terrasse, 91190 Gif-sur-Yvette, France; PhuongY.MAI@cnrs.fr (P.-Y.M.); Geraldine.LEGOFF@cnrs.fr (G.L.G.); Philippe.LOPES@cnrs.fr (P.L.); 2 Équipe “Chimie des Substances Naturelles” BioCIS, CNRS, Université Paris-Saclay, 5 Rue J.-B. Clément, 92290 Châtenay-Malabry, France; erwan.poupon@universite-paris-saclay.fr (E.P.); mehdi.beniddir@universite-paris-saclay.fr (M.A.B.); 3 PACIFIC BIOTECH SAS, BP 140 289, 98 701 Arue, Tahiti, French Polynesia; xmoppert@pacific-biotech.pf (X.M.); bcosta@pacific-biotech.pf (B.C.)

**Keywords:** solid-phase extraction SPE, XAD resin, molecular networking, *Streptomyces*, specialized metabolites, dereplication

## Abstract

Solid-phase extraction embedded dialysis (SPEED technology) is an innovative procedure developed to physically separate in-situ, during the cultivation, the mycelium of filament forming microorganisms, such as actinomycetes and fungi, and the XAD-16 resin used to trap the secreted specialized metabolites. SPEED consists of an external nylon cloth and an internal dialysis tube containing the XAD resin. The dialysis barrier selects the molecular weight of the trapped compounds, and prevents the aggregation of biomass or macromolecules on the XAD beads. The external nylon promotes the formation of a microbial biofilm, making SPEED a biofilm supported cultivation process. SPEED technology was applied to the marine *Streptomyces albidoflavus* 19-S21, isolated from a core of a submerged Kopara sampled at 20 m from the border of a saltwater pond. The chemical space of this strain was investigated effectively using a dereplication strategy based on molecular networking and in-depth chemical analysis. The results highlight the impact of culture support on the molecular profile of *Streptomyces albidoflavus* 19-S21 secondary metabolites.

## 1. Introduction

Specialized metabolites (also known as secondary metabolites) produced by living organisms represent an inexhaustible source of molecules of biological interest to solve current and future public health challenges. This area of research, especially in the field of metabolites from micro-organisms, was accelerated by the development of genome-based technologies [1,2]. The era of drug lead discovery from culturable bacteria is not nearing its end, however its success relies on researchers’ ability to innovate in strategies used to collect samples from the environment [3] and methods used to trap specialized metabolites from culture media [4]. Nevertheless, one of the major persisting drawbacks is the isolation of sufficient quantities of compounds to carry out chemical and biological investigations.

Among various concentration techniques, Amberlite XAD resin trapping of organic compounds gained recent interest and finds application in plant [5,6] marine invertebrate [7,8], and microbial [9,10,11,12,13,14,15,16,17,18] specialized metabolite extraction.

We have recently extended the exploitation of in-situ XAD-16 extraction of mycelium forming microorganisms, actinomycetes and fungi [9,10,11,12,13,14,15,16,17,18], known as the major providers of valuable bioactive compounds [19,20]. Compared to submerged cultivation, we showed that in-situ XAD-16 extraction significantly impacts the diversity and the yields of target compounds [9,18].

**Figure 1:**
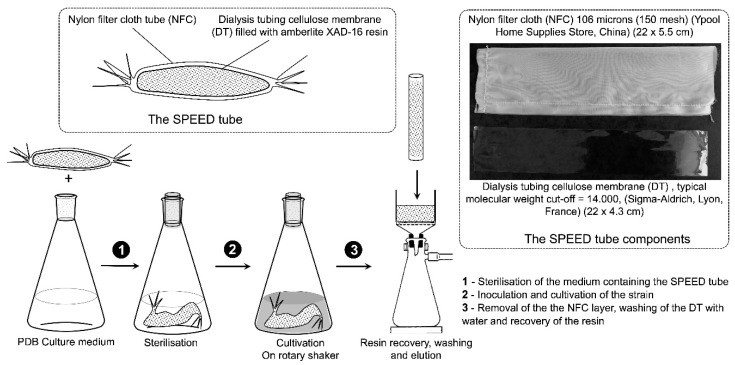
Schematic representation of SPEED technology.

Although this approach strongly facilitates the extraction of target products with considerable savings in the use of organic solvents, application of in-situ XAD-16 extraction to mycelium forming micro-organisms leads to significant entrapment of the resin beads into the filamentous network, and complicates the separation of these two compartments. As a consequence, during the elution step of the resin/mycelium mixture by appropriate solvents, the analytical profile is often contaminated with undesired compounds, which compromises the purification steps. The solid-phase extraction embedded dialysis (SPEED technology) is based on a physical separation between the microbial biomass and the resin used for in-situ solid phase extraction. It includes a two-layer barrier consisting of an external nylon filter cloth (NFC) and an internal dialysis tube (DT) containing the resin beads. According to the molecular cut of the DT, only molecules with appropriate molecular weight can flow from the cultivation broth to the resin. This selectivity added to the ease of recovering clean resin beads and eluate, makes SPEED technology an indisputable added value to the study of microbial specialized metabolites. The SPEED technology is reported in this paper for the first time and summarized in Figure 1.

**Figure 2:**
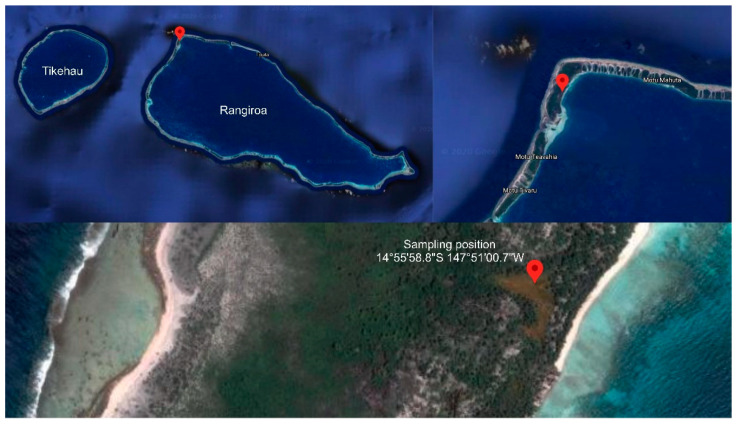
Kopara sampling location reported in this work.

In order to develop and optimize SPEED technique, a case-study was needed. An ongoing project from our consortium appeared to provide an ideal framework towards this end. In our efforts to isolate and investigate micro-organisms from under-explored ecosystems, samples from microbial mats called ‘Kopara’ were collected from Rangiroa atoll, in French Polynesia. Kopara mats were previously described as vertically organized cyanobacteria strata of 20 to 50 cm thickness. The Kopara geomorphology, physico-chemistry and microbial diversity were previously reported [21,22,23], however only bacterial pigments and exopolymers (exopolysaccharides and poly-β-hydroxyalkanoates) were studied and were shown to be secreted by some Kopara microbial isolates [24,25]. Our running program is dedicated to the isolation, for the first time, of filament-forming microorganisms, actinomycetes and fungi, from Kopara samples. To do so, 56 Kopara samples and 37 other materials (water, animals) from different locations of Rangiroa atoll at different depth from surface to −20 cm or more were collected (Figure 2).

Facing a large number of samples and to avoid the rediscovery of known compounds, which is the major issue in natural product chemistry, the immediate identification of known compounds and prioritization of interesting strains that produce potential new compounds deserve intense effort. To meet this challenge, the dereplication strategy using metabolomics profiling based on mass spectrometry has been employed to perform the potential interesting strain screening program. Indeed, molecular networking approach allows the organization of untargeted tandem mass spectrum datasets according to their spectral similarity and generates clusters of structurally related metabolites. This approach has become a powerful tool for navigating the chemical space of complex biological systems and can be used to view the chemical constituents of a wide variety of extracts in a single map [26].

## 2. Results and Discussions

### 2.1. Collection Site and Strain Identification

**Figure 3:**
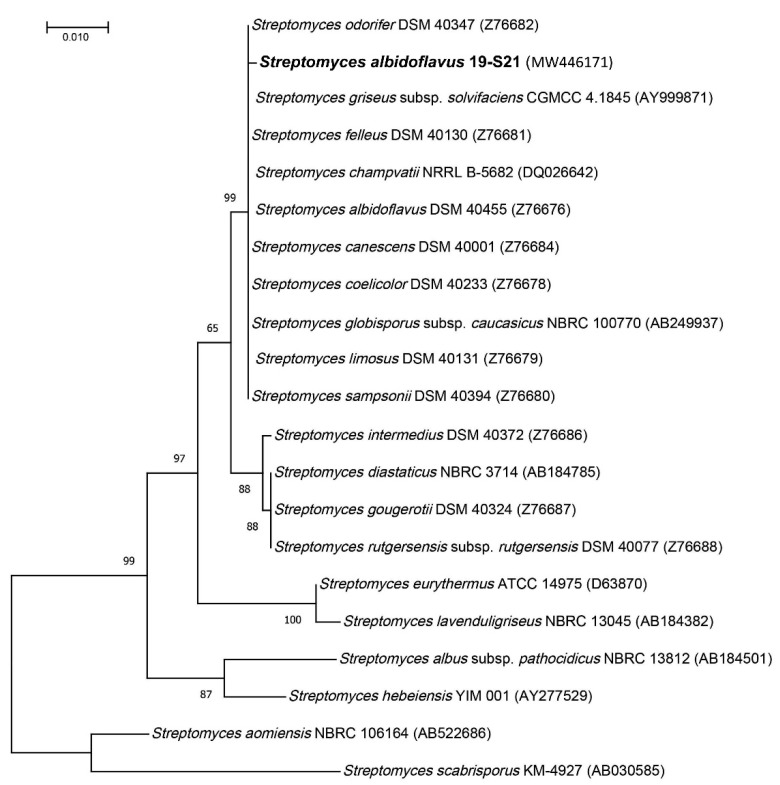
Maximum-likelihood tree obtained from 16S rRNA sequence alignment of the isolate and *Streptomyces* spp. of the *albidoflavus* group of species and close relatives selected from [28]. Bootstrap values are reported as percentages (1000 replicates). GenBank^®^ accessions are mentioned between brackets.

Starting from the Kopara sample (see Section 4), the stain *Streptomyces albidoflavus* 19-S21 was isolated and purified by serial inoculation on Potatoes Dextrose Agar slants. From the phylogenetic analysis based on the 16S rRNA sequence, the isolate was found in the *albidoflavus* group (Figure 3). Since all of the species in this clade (*S. canescens*, *S. champvatii*, *S. coelicolor*, *S. felleus*, *S. globisporus* ssp *caucasicus*, *S. griseus* ssp. *solvifaciens*, *S. limosus*, *S. odorifer*, *S. sampsonii*) were previously classified as heterotypic synonyms of *S. albidoflavus* [27], we have named the isolate *Streptomyces albidoflavus* 19-S21 with the GenBank^®^ accession number MW446171.

### 2.2. S. albidoflavus 19-S21 Cultivation according to SPEED Technology

As described above, the main advantage of SPEED technology is the physical separation of the strain mycelium and the XAD-16 resin beads, used for in-situ SPE. The DT barrier is a second advantage as it discriminates between large biomolecules and secondary metabolites. The 1.4 kD cut-off of the dialysis membrane guarantees permeability to all families of specialized metabolites [29].

**Figure 4:**
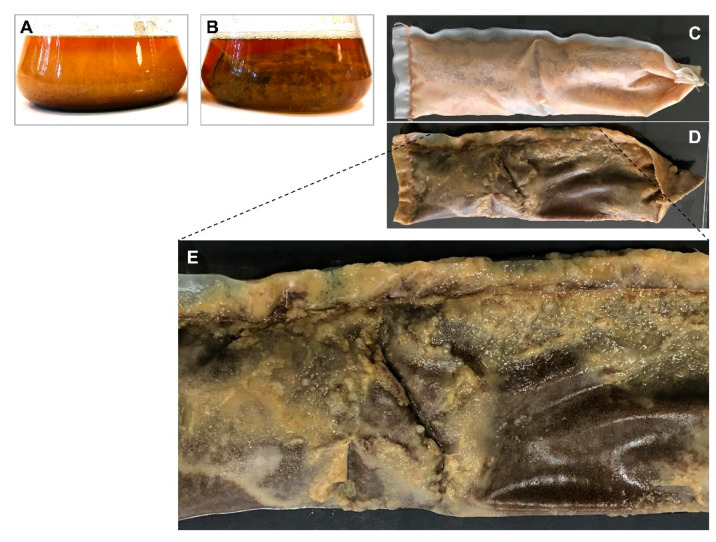
Difference between submerged and SPEED cultivation of *S. albidoflavus* 19-S21 in PDB medium: (**A**) 11 days submerged cultivation with in-situ SPE with XAD-16 resin; (**B**) 11 days SPEED cultivation; (**C**) SPEED tube after removal of the biofilm layer; (**D**) SPEED tube with the biofilm layer; (**E**) Focus on D showing the biofilm layer attached to the NFC.

Beyond these substantial improvements, we made an unexpected and intriguing observation which was not only reproducible for the strain *S. albidoflavus* 19-S21, but also for other actinomycetes and fungi (S11). Thus, compared to the submerged cultivation in which the mycelium/resin mixture is homogenously spread in the medium (Figure 4A), under SPEED condition, the mycelium formed a dense and stable biofilm attached to the external nylon filter cloth (NFC), with almost no mycelium floating in the medium (Figure 4B). When the SPEED tube was recovered, the biofilm remained sticking to the NFC (Figure 4D,E), and was removed by gentle scraping under running water, before removing the dialysis tube (DT). Therefore, SPEED technology cannot be assimilated to a classical submerged cultivation as the biomass adopts a biofilm type organization.

**Figure 5:**
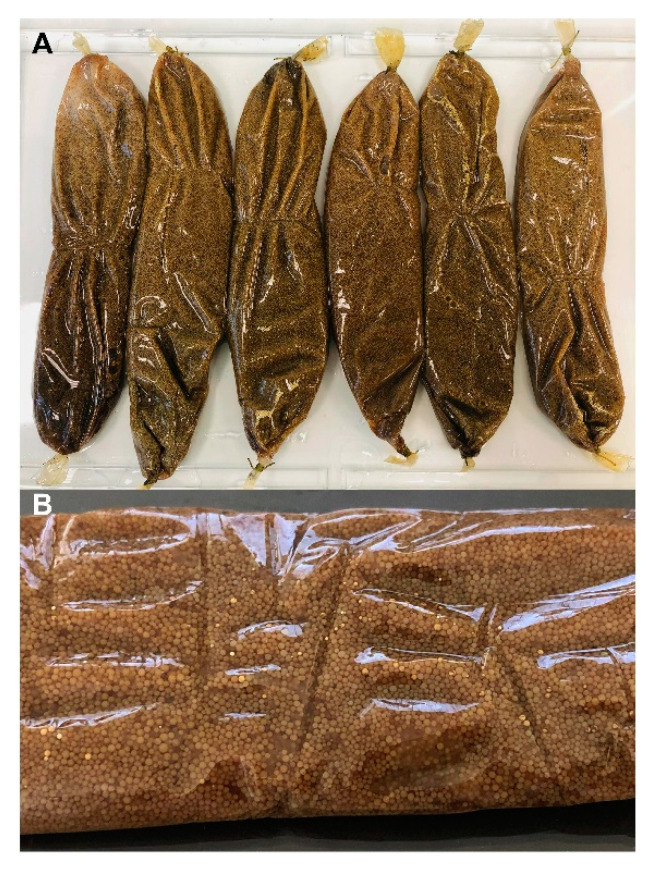
Dialysis tubes (DT) recovery: (**A**) after SPEED cultivation of *S. albidoflavus* 19-S21 in PDB medium; (**B**) Focus showing colored resin inside the DT.

At the end of the SPEED cultivation period and the removal of the NFC, the DTs were recovered easily and cleanly (Figure 5). The subsequent steps consist of the classical recovery of the resin and metabolite elution as reported in Section 4.

Microbial biofilm association to nylon nets and cloth is well documented, mainly in the aquaculture context [30] and treatment of polluted water [31]. Nylon was also used to support and promote algal biofilm growth [32].

### 2.3. Molecular Networking-based Chemical Exploration of S. albidoflavus 19-S21 Specialized Metabolites

The strain was cultivated in different conditions (see Section 4). Resins and media were extracted with ethyl acetate and methanol consequently. In LSF, insignificant quantity and diversity of metabolites were observed. This was also the case for all methanol extracts. On the other hand, the ethyl acetate extracts of the resins showed various metabolic HPLC profiles. In order not to miss any compound, all ethyl acetate and methanol extracts of the resins were analyzed.

SPEED extracts were submitted to UPLC-MS/MS profiling and the resulting data were processed following the feature-based molecular networking workflow [33]. The global molecular network was color-tagged according to multiple culture conditions and the solvents used for the extraction. The MS/MS data were annotated using the GNPS spectral library (Figure S1) [34]. The network consists of 2714 nodes, consisting of 136 clusters and regrouping nodes with related structures.

All library hits resulting from GNPS dereplication are listed in Table S1 in Supporting Information. The nodes with annotation are also visualized in Cytoscape^®^ 3.7.2 [35] and filled by different color codes to easily distinguish the dereplicated nodes from the non-dereplicated ones (Figure S2).

**Figure 6:**
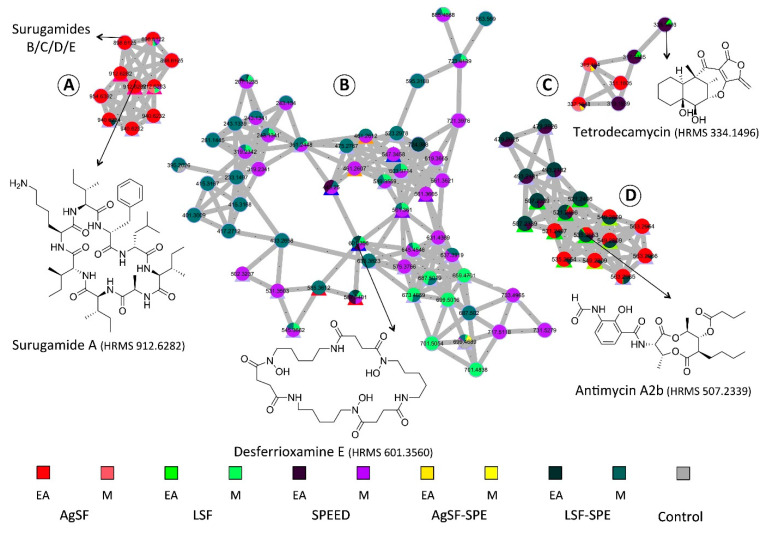
The molecular network built from different crude extracts of the strain *Streptomyces albidoflavus*. Some clusters with annotation are highlighted in this figure: (**A**) Cluster of surugamide family; (**B**) Cluster related to desferrioxamines; (**C**) Antimycin-type depsipeptide clusters; and (**D**) Unannotated cluster mainly produced in SPEED culture condition. In this figure, EA stands for ethyl acetate and M stands for methanol. The control condition represents different ethyl acetate and methanol extracts of resin XAD from different culture conditions and also those of PDB agar, which were not inoculated by the strains.

According to the global molecular network, the strain specialized metabolites varied according to the different culture conditions. One cluster of surugamides (Figure 6A) has been found to be produced only in solid culture Surugamides belonging to a known family of cyclic octapeptides, initially isolated from a Marine *Streptomyces* sp. JAMM992 and has also been proved to possess anticancer and antifungal properties [36]. One cluster of desferioxamines (Figure 6B), which are siderophores produced by bacteria, has been found mainly in methanol extracts and produced by the strain in liquid culture condition. Antimycin A1 along with three other antimycins (antimycins A2, A3, and A4) were annotated and produced by the strain in different culture conditions (Figure 6C). Antimycins include various scaffolds due to the differences in the size of the lactone and its substitution patterns. These compounds have been described for many *Streptomyces* sp. and exhibited interesting biological activities, such as antifungal, insecticidal, nematocidal, and piscicidal activities, because of their ability to block the electron transport in mitochondria. Several antimycin classes have also been reported to possess potent anti-inflammatory and antitumoral activities [37,38].

One unannotated cluster (D in Figure 6) has been detected on the global molecular network. A compound in this cluster at *m/z* = 335.1459, attracted our attention because of its salient production in SPEED culture condition (node encircled in red color in Figure 6). As a way to dereplicate this node, the molecular formula related to its exact mass was generated and then searched against the AntiBase^®^ database (Wiley) and the Dictionary of Natural Products^®^. The database search yielded 125 hits with only one molecule, tetrodecamycin, being reported from the species *Streptomyces nashvillensis* MJ885-mF8 [39]. All the 125 hit compounds and their biological source are listed in Table S2.

### 2.4. Isolation of Representative Compounds

**Figure 7:**
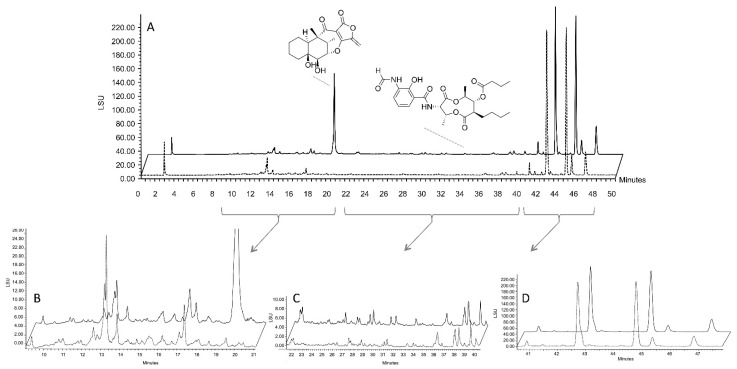
Comparison of HPLC profiles under SPEED and LSF-SPE culture conditions: (**A**) HPLC-ELSD analysis of the SPEED (continuous line) and LSF-SPE (dashed line). According to the UV spectrum of compounds we can delimit three zones: (**B**) containing mainly tetrodecamycin; (**C**) zone containing mainly antimycin-type depsipeptides; and (**D**) fatty acid zone.

In order to confirm HRMS-based dereplication of the compounds at *m/z* = 335.1459 along with the annotations provided by the GNPS, we used the resin extract to purify the representative compounds of major molecular node. *S. albidoflavus* 19-S21 was cultivated according to two procedures involving XAD-16 in-situ extraction during the culture; LSF-SPE [9] and SPEED technology. The resins were extracted by ethyl acetate followed by methanol, and the aqueous filtrated medium was concentrated under vacuum. These three fractions were analyzed by thin layer chromatography (TLC) and HPLC confirming that almost all the formed metabolites were recovered in the ethyl acetate extract. Figure 7 represents a superposition of the ELSD chromatograms of LSF-SPE (dashed line) and SPEED extracts (continuous line). One of the major compounds at 20.2 min is produced only in SPEED condition (Figure 7A,B). The molecular formula was established as C_18_H_23_O_6_ based on its HR-ESIMS data ([M + H]^+^ at 335.1496) (Figure S5). The compound was identified as tetrodecamycin by comparison of its ^1^H and ^13^C NMR data with those reported in literature (Figures S6 and S7) [39]. The other major non-polar compounds eluted after 40 min were also investigated. These compounds were characterized as fatty acids by comparison with literature [40]. The experimental data of 14-methylpentadecanoic acid, which is one of the fatty acids, were mentioned in the Supplementary Information (Figures S8–S10). The compounds between 22 and 40 min belong to the antimycin-type depsipeptides. To demonstrate the performance of the dereplication approach based on molecular networking, compound with *m/z* 507.2339 (Figure 6C and Figure 7A) was further isolated (Figure 6C) and fully characterized. The structural data are reported in the experimental section and compared to literature as actinomycin A4a (Figures S3 and S4) [41,42].

## 3. Discussion

Molecular networking offers the possibility to map additional information, such as biological, analytical, and taxonomic details over networks [43]. Hence, the strain *Streptomyces albidoflavus* 19-S21 was prioritized for further study based on its chemical originality after examination of a multi-informative annotated global molecular network. This strain was isolated from a core of a submerged Kopara, sampled at 20 m from the border of a saltwater pond. The strain was cultivated in Potatoes Dextrose Broth (PDB) under different conditions including SPEED technology.

SPEED technology, as disclosed in this paper, opens a new area in microbial cultivation. The biofilm formed could be assimilated to a solid culture which may explain the difference in the formed metabolites compared to LSF-SPE liquid culture. It is well documented that the life cycle of filamentous micro-organisms, actinomycetes and fungi, is drastically impacted by the cultivation support; and that the regulators of cell cycle phases impacts in parallel the expression of specialized metabolites clusters [44,45]. The life cycle including filament, sexual organs, and fructifications production takes place naturally on living supports like roots, bark, and leaves in plants, or on rocks, soil, or dead wood. In the marine ecosystem, these microorganisms are mainly associated to sponges and corals [46,47].

In plants, the formation of biofilm provides major advantages to symbionts and impacts the strain metabolome [48,49]. Recent attempts have been reported in the literature, aiming at culturing marine microorganisms on a cotton scaffold [50]. Microbial colonies were formed on the cotton fibers and the metabolites profile was significantly impacted. In SPEED procedure, the molecular cut-off discrimination of the internal dialysis tube, and the biofilm-like growth on the external nylon cloth are the main advantages. The XAD resin inside the dialysis tube allows the solid phase extraction of metabolites below the dialysis molecular cut, and the nylon tissue pores separate physically the mycelium from the resin, which made in-situ SPE very easy to handle and the biomass to grow as this sticky and dense biofilm. The next step is to convert this proof of concept to a technological device, allowing the scale-up and automation of the experiments.

## 4. Materials and Methods

### 4.1. Strain Isolation

In September 2018, a Kopara sample was collected from a core of a submerged Kopara mat located at 20 m from the border of a saltwater pond at the Rangiroa atoll, French Polynesia (Sampling coordinates 14°55′58.8′′ S 147°51′00.7′′ W). The Kopara sample was ground and homogenized in sterile water and decanted. The suspension was serially diluted, plated on PDB agar slants, and incubated at 28 °C for 1 to 6 weeks. The strain was cultivated in Potatoes Dextrose Broth (PDB Difco, Fisher Scientific, Illkirch, France). The stain *Streptomyces albidoflavus* 19-S21 was isolated and purified by serial inoculations on Potatoes Dextrose agar slants.

### 4.2. Phylogeny Investigation

Genomic DNA isolation and amplification of the ITS region was performed as described previously [51]. Amplicons were sequenced by Sanger sequencing and the sequences were aligned against the 16S ribosomal RNA database of the Targeted Loci project of NCBI using MUSCLE. The alignment was manually inspected and gaps were removed. The evolutionary history was inferred by using the maximum likelihood method and Tamura-Nei model [52] with 1000 replicates. The initial tree for the heuristic search was obtained automatically by applying Neighbor-Join and BioNJ algorithms to a matrix of pairwise distances estimated using the maximum composite likelihood (MCL) approach, and then selecting the topology with superior log likelihood value. Evolutionary analyses were conducted in MEGA X version 10.1.7 [53].

### 4.3. Strain Cultivation with In-Situ SPEED Technology

*Streptomyces albidoflavus* 19-S21 mycelium was conserved at −20 °C in 20% glycerol and was revived for 5 days on a 3 × 15 cm Petri plates (NUNC DISH 150 × 10, Thermo Fisher Scientific, Les Ulis, France) containing PDB agar (Difco, Thermo Fisher Scientific). For liquid cultivation, sterile water was poured onto the plates (16 mL per plate), and the mycelium recovered by gentle scratching of the surface with a scalpel. Then, 10 × 2 L Erlenmeyer containing 1 L of PDB medium and a SPEED tube before sterilization (Figure 1) were inoculated. Each DT tube was filled with 40 g of XAD-16 resin (AMBERLITE^®^ XAD^®^16HP N, DOW France SAS, Saint-Denis, France). The mycelium suspension was then introduced in each Erlenmeyer (4 mL) and the strain cultivated for 13 days under stirring (130 rpm, 28 °C). After cultivation, the SPEED tubes were taken out from the Erlenmeyer, rinsed thoroughly under running water to remove the sticking biomass. The external NFC tube was removed and the DT recovered and washed back under running water. The XAD resin was then recovered from the DT, placed in a Büchner funnel, washed extensively with water, then dried under vacuum to remove the residual water. Then, 425 g of XAD-16 resin was recovered from the 10 L cultivation and was submitted to the extraction steps. The strain was cultivated in 5 conditions:

- Agar-state fermentation (AgSF);
- Liquid-state fermentation (LSF);
- SPEED cultivation;
- Agar-state fermentation coupled to SPE (AgSF-SPE) [11];
- Liquid-state fermentation coupled to SPE (LSF-SPE).

### 4.4. Extraction/Purification Procedures

The 425 g of XAD-16 resin were transferred in a 2 L glass bottle (Duran) and the trapped compounds gently eluted with 3 × 1 L of ethyl acetate (4 h per extraction). The extracts were pooled, dried on anhydrous sodium sulfate, and evaporated under reduced pressure to offer 577 mg of extract. The resin is extracted back with 2 × 1 L of methanol (4 h per extraction) and the methanol evaporated leading to 1.15 g of extract.

The classical purification procedure involves a flash chromatography step followed by semi-preparative HPLC purification of the flash chromatography fractions.

### 4.5. Characterization, Isolation, and Structural Elucidation Experiments

The analytical HPLC system consisted of an Alliance Waters 2695 controller coupled with a PhotoDiode Array detector Waters 2996 (UV), an evaporative light-scattering detector (ELSD) Waters 2424 detector and a mass detector Waters QDa (MS) (Waters SAS, Saint-Quentin-en-Yvelines, France). A Sunfire C18 column (4.6 × 150 mm, 3.5 μm) was used with a flow rate of 0.7 mL/min. The elution gradient consisted of a linear gradient from 100% solvent A to 100% solvent B in 40 min, then 10 min at 100% B (Solvent A: H_2_O + 0.1% HCOOH, Solvent B: ACN + 0.1% HCOOH). Preparative HPLC was performed using the same gradient on a semi-preparative Sunfire C_18_ column (10 × 250 mm, 5 μm) using a Waters autosampler 717, a pump 600, a photodiode array detector 2996, and an ELSD detector 2420 (Waters SAS, Saint-Quentin-en-Yvelines, France). Pre-packed silica gel Redisep columns were used for flash chromatography using a Combiflash-Companion chromatogram (Serlabo, Entraigues-sur-la-Sorgue, France). All other chemicals and solvents were purchased from SDS (SDS, Peypen, France). NMR experiments were performed using a Bruker Avance III 600 MHz spectrometer equipped with a TCI cryo-probe head, and a Bruker Avance 500 MHz spectrometer (Bruker, Vienna, Austria). The spectra were acquired in CD_3_OD (δ_H_ 3.31 ppm and δ_C_ 49.15 ppm), in CDCl_3_ (δ_H_ 7.26 ppm and δ_C_ 77.16 ppm).

### 4.6. Data Dependent LC-ESI-HRMS2 Analysis

UPLC-ESI-HRMS^2^ analyses were achieved by coupling the UPLC system to a hybrid quadrupole time of-flight mass spectrometer Agilent 6546 (Agilent Technologies, Massy, France) equipped with an ESI source, operating in both positive and negative ion mode.

A ZORBAX^®^ Eclipse Plus C18 UPLC column (2.1 × 50 mm; i.d. 1.8 μm, Agilent) was used, with a flow rate of 0.5 mL·min^–1^ and a linear gradient from 5% B (A: H_2_O + 0.1% formic acid, B: Acetonitrile + 0.1% formic acid) to 100% B over 15 min. Source parameters were set as followed: capillary temperature at 320 °C, source voltage at 3500 V, sheath gas flow rate at 11 L·min^–1^. The divert valve was set to waste for the first 3 min. MS scans were operated in full-scan mode from *m/z* 100 to 1200 (0.1 s scan time) with a mass resolution of 67,000 at *m/z* 922. A MS^1^ scan was followed by MS2 scans of the five most intense ions above an absolute threshold of 3000 counts. Selected parent ions were fragmented at a collision energy fixed at 35 eV and an isolation window of 1.3 amu. In the positive-ion mode, purine C_5_H_4_N_4_ [M + H]^+^ ion (*m/z* 121.050873) and the hexakis (1H,1H,3H-tetrafluoropropoxy)-phosphazene C_18_H_18_F_24_N_3_O_6_P_3_ [M + H]^+^ ion (*m/z* 922.009 798) were used as internal lock masses. In the negative-ion mode, trifluoroacetic acid (CF_3_CO_2_H, *m/z* 112.98559) and the trifluoroacetate adduct with *m/z* 1033.988109 were used. A permanent MS/MS exclusion list criterion was set to prevent oversampling of the internal calibrant. LC-UV and MS data acquisition and processing were performed using MassHunter^®^ Workstation software (Agilent Technologies, Massy, France).

### 4.7. MS Data Processing and Feature-Based Molecular Networking—GNPS

The MS2 data files, related to the 21 extracts were converted from the .d (Agilent) standard data-format to .mzML format using the MSConvert software, part of the ProteoWizard package [54]. All .mzML were then processed using MZmine 2v53 [55]. The mass detection was realized keeping the noise level at 10,000 at MS1 level and at 1000 at MS2. The ADAP chromatogram builder was used using a minimum group size of scans of 2, a group intensity threshold of 3000, a minimum highest intensity of 3.0E3 and *m/z* tolerance of 0.005 *m/z* or 50 ppm. The chromatogram deconvolution was performed using the Wavelets (ADAP) with the following settings: *m/z* range for MS2 scan pairing (Da) = 0.06, RT range for MS2 scan pairing (min) = 1, S/N threshold = 5, S/N estimator = Intensity window SN, min feature height = 3000, coefficient/area threshold = 2, Peak duration range = 0.00–0.90 and RT wavelet range = 0.00–0.09 [56]. Isotopes were grouped using the isotopic peaks grouper algorithm with an *m/z* tolerance of 5 ppm and a RT tolerance of 0.2 min with the most intense peak. The peak alignment algorithm was used with the following settings: *m/z* tolerance of 0.004 or 5 ppm, weight for *m/z* of 1, retention time tolerance of 0.1, and weight for RT of 1. The resulted peak list was filtered to keep only rows with MS2 features. The .mgf and .csv (for RT, *m/z*, peak areas) files were exported using the dedicated “Export/Submit to GNPS/FBMN” option. The raw data files related to the LC-MS/MS analysis of the fractions were deposited on the public MassIVE repository under the accession number: MSV000087546. The MS/MS spectrum of tetrodecamycin was deposited in the GNPS spectral library under the identifier: CCMSLIB00006581621.

### 4.8. Molecular Networking Parameters

A molecular network was created using the online FBMN workflow (version release_27) at GNPS (http://gnps.ucsd.edu, accessed on 7 March 2021) with a parent mass tolerance of 0.02 Da and an MS/MS fragment ion tolerance of 0.02 Da. A network was then created where edges were filtered to have a cosine score above 0.6 and more than 3 matched peaks. Further edges between two nodes were kept in the network if and only if each of the nodes appeared in each other’s respective top 10 most similar nodes. The spectra in the network were then searched against GNPS spectral libraries. All matches kept between network spectra and library spectra were required to have a score above 0.6 and at least 4 matched peaks. The analog search has also performed. The molecular networking data were analyzed and visualized using Cytoscape^®^ (ver. 3.7.2) [36].

## Data Availability

Data are available from the corresponding author.

## Supplementary Materials

The following are available online at https://www.mdpi.com/article/10.3390/md19070371/s1.

## Abbreviations

GNPS: Global Natural Product Social Molecular Networking; FBMN: Feature-Based Molecular Networking; ADAP: Automated Data Analysis Pipeline; MassIVE: Mass Spectrometry Interactive Virtual Environment; PDA: Photo-diode detector; LSD: light scattering detector; RT: retention time; S/N: signal/noise.
