# Supplementary material for: Solid-Phase Extraction Embedded Dialysis (SPEED), an Innovative Procedure for the Investigation of Microbial Specialized Metabolites
Source: Mar Drugs. 2021 Jun 26;19(7):371. doi: 10.3390/md19070371 (PMC8304039; doi:10.3390/md19070371)
Supplement: Supplementary file 1 [file marinedrugs-19-00371-s001.zip › marinedrugs-1280839-supplementary.pdf]

Solid-Phase Extraction Embedded Dialysis (SPEED) an Innovative procedure for the Investigation of Microbial Specialized Metabolites

Phuong-Y MAI , Géraldine Le Goff, Erwan Poupon , Philippe Lopes , Xavier Moppert, Bernard Costa , Mehdi A. Beniddir, Jamal Ouazzani *

Supporting Information

Table

Table S1: Table of hit compounds dereplicated by GNPS database

Table S2 The hit list from the Dictionary of Natural Products® of 125 hit compounds of the molecular formula $C_{18}H_{22}O_6$ and their biological source

Figures

Figure S1: The molecular networking of the strain extracts from different condition cultures and the control extracts. Different culture condition is represented by different color code.
Figure S2: The molecular networking with dereplicated nodes that are mapped by rainbow color codes while others stayed purple.
Figure S3: The HRESIMS spectrum of antimycin A4a
Figure S4: ^1H NMR spectrum of antimycin A4a in $CDCl_3$ (500 MHz)
Figure S5: The HRESIMS spectrum of tetrodecamycin
Figure S6: ^1H NMR spectrum of tetrodecamycin in $CDCl_3$ (500 MHz)
Figure S7: ^{13}C NMR spectrum of tetrodecamycin in $CDCl_3$ (125 MHz)
Figure S8: The HRESIMS spectrum of 14-methylpentadecanoic acid
Figure S9: ^1H NMR spectrum of 14-methylpentadecanoic acid in $CDCl_3$ (500 MHz)
Figure S10: ^{13}C NMR spectrum of 14-methylpentadecanoic acid in $CDCl_3$ (125 MHz)
Figure S11. Further SPEED pictures

Table S1. Table of hit compounds dereplicated by GNPS database.

	Compound name	Score	Biological source
912.6279	Surugamide A	0.78	Marine-derived *Streptomyces* sp[1]
623.3375	Desferrioxamine E	0.89	*Streptomyces* species[2]
614.2724	Ferrioxamine B	0.76	Marine bacterium[3]
601.3555	Desferrioxamine E	0.84	*Streptomyces* species[2]
587.3401	Desmethylenylnocardamine	0.83	*Streptomyces* species[4]
585.3606	Dehydroxynocardamine	0.83	*Streptomyces* species[5]
561.3606	Desferrioxamine	0.92	*Streptomyces pilosus* ATCC 19797 [6]
549.2809	Antimycin A1	0.81	*Streptomyces* sp[7]
547.3451	N-[5-[[4-[4-[acetyl(hydroxy)amino]butylamino]-4-oxobutanoyl]-hydroxyamino]pentyl]-N'-(5-aminopentyl)-N'-hydroxybutanediamide	0.87	-
531.4093	Didodecyl 3,3'-thiodipropionate oxide	0.71	
535.2652	Antimycin A2	0.83	*Streptomyces* sp[7]
521.2498	Antimycin A3	0.83	*Streptomyces* sp[7]
507.2338	Antimycin A4	0.75	*Streptomyces* sp[7]
461.2607	4-[5-[[4-[5-[acetyl(hydroxy)amino]pentylamino]-4-oxobutanoyl]-hydroxyamino]pentylamino]-4-oxobutanoic acid	0.85	-
443.2500	4-[5-[[4-[5-[acetyl(hydroxy)amino]pentylamino]-4-oxobutanoyl]-hydroxyamino]pentylamino]-4-oxobutanoic acid	0.74	-
425.1919	Citreoviridin	0.72	*Penicillum citreoviride*[8]
369.1252	Tricresylphosphate	0.86	-
367.1501	1-beta-D-Glucopyranosyl-L-tryptophan	0.85	-
314.09	5'-Deoxy-5'-(methylsulfinyl)adenosine	0.86	-
261.1234	Cyclo(L-Tyr-L-Pro)	0.83	*Streptomyces* sp[9]
245.1286	L-prolyl-L-phenylalanine	0.80	-
188.0706	L-Tryptophan	0.91	-
174.0551	Quinoline-4-carboxylic acid	0.77	-
169.0756	Norharman	0.95	Plant and fungi metabolite[10]

Table S2 The *hit list* from the Dictionary of Natural Products® of 125 hit compounds of the molecular formula $C_{18}H_{22}O_6$ and their biological source

No.	compound name	biological source
1	aigialomycin D	*Aigialus parvus* BCC 5311 [11], *Fusarium* sp. LN-10 and *Paecilomyces* sp. SC0924
2	aigialomycin E	*Aigialus parvus* BCC 5311 [11]
3	2,2′,4,4′,5,5′-hexamethoxybiphenyl	-
4	2,2′,4,4′,6,6′-hexamethoxybiphenyl	-
5	1,2-Bis(4-hydroxy-3-methoxyphenyl)-1,3-propanediol;(1R,2R)-form, 1-Me ether	*Phyllanthus glaucus* [12]
6	1,2-Bis(4-hydroxy-3-methoxyphenyl)-1,3-propanediol;(1R,2S)-form, 1-Me ether	*Phyllanthus glaucus* [12]
7	1,2-Bis(4-hydroxy-3-methoxyphenyl)-1,3-propanediol;(1RS,2SR)-form, 1-Me ether	*Phyllanthus glaucus* [12]
8	1,2-Bis(4-hydroxy-3-methoxyphenyl)-1,3-propanediol;(1S,2R)-form, 1-Me ether	*Phyllanthus glaucus* [12]
9	1,2-Bis(4-hydroxy-3-methoxyphenyl)-1,3-propanediol;(1S,2S)-form, 1-Me ether	*Phyllanthus glaucus* [12]
10	1,2-Bis(3,4,5-trihydroxyphenyl)ethane; 3,3',4,4'-Tetra-Me ether	*Dendrobium cumulatum [13]*
11	dihydrobyssochlamic acid	*Byssochlamys fulva* [14]
12	chaetoaurin	*Chaetomium aureum* [15]
13	chermesinone C	*Penicillium chermesinum* ZH4-E2 [16]
14	combretastatin	*Combretum caffrum* [17]
15	cyclopaldic acid, 5-*O*-(3-methyl-2-butenyl), 3-Et ether	*Aspergillus duricaulis* [18]
16	3,7'-cyclo-1,2,3,4-tetrahydro-1,8'-lign-8-ene-2,3,3',4,4',5-hexol	-
17	3,7'-Cyclo-1,2,3,4-tetrahydro-1,8'-lign-8-ene-2,3,3',4,4',5-hexol; (1*R**,3*S**,4*R**,7'*R**,8'*S**)-form	-
18	3,7'-Cyclo-1,2,3,4-tetrahydro-1,8'-lign-8-ene-2,3,3',4,4',5-hexol; (1*R**,3S*,4*S**,7'*R**,8'*S**)-form	-
19	3,7'-Cyclo-1,2,3,4-tetrahydro-1,8'-lign-8-ene-2,3,3',4,4',5-hexol; (1*S*,2*S*,3*R*,4*S*,7'*S*,8'*R*)-form	-
20	cytosporone H	*Paraphaeosphaeria quadriseptata* [19]
21	cytosporone G	*Paraphaeosphaeria quadriseptata* [19]
22	cytosporone I	*Paraphaeosphaeria quadriseptata* [19]
23	Neocosmosin B	*Neocosmospora* sp. (UM-031509) [20]
24	Monocillin V	*Monocillium nordinii* [21]
25	9R-Hydroxymonocillin IV	*Paecilomyces sp. SC0924* [22]
26	3,5-Dihydroxy-4(15),10(14),11(13)-guaiatrien-12,8-olide; (1α,3α,5α,8α)-form, 10α, 146epoxide, 3-propanoyl	*Arctotis grandis* and *Venidium hirsutum*
27	3,5-Dihydroxy-4(15),10(14),11(13)-guaiatrien-12,8-olide; (1α,3β,5α,8α)-form, 10α, 146epoxide, 3-propanoyl	*Arctotis arctotoides*
28	1,8-Dihydroxy-2-oxo-3,7(11)-eudesmadien-12,8-olide; (1β,8βOH)-form, 8-Me ether, 1-Ac	*Salvia plebeia*
29	7-hydroxygerin	*Saussurea cauloptera* [23]

30	carayensin A	*Carya cathayensis* [24]
31	2-(3,4-Dihydroxyphenyl)-1-(3,4,5-trihydroxyphenyl)ethanol; *(R)*-form, α,3,3′,5-Tetra-Me ether (loddigesiinols C)	*Dendrobium loddigesii* [25]
32	nobilin B	*Dendrobium nobile* [26]
33	pithecellobiumol A	*Pithecellobium clypearia* [27]
34	combretol C	*Combretum yunnanense* [28]
35	3,8-dimethyl-2-naphthol; *O*-β-D-glucopyranoside	*Hibicus cannabinus* [29]
36	8,10-epoxy-*p*-mentha-1,3,5-triene-2,5,9-triol; 2-Me ether, 5-tigloyl, 9-Ac	*Mikania species* [30]
37	4,7'-epoxy-3,4,5,6-tetrahydro-3,8'-lign-8-ene-3,3',4,4',6-pentol	-
38	4,7'-epoxy-3,4,5,6-tetrahydro-3,8'-lign-8-ene-3,3',4,4',6-pentol; (3*R**,4*R**,7′S*,8′*S**)-form	-
39	4,7'-epoxy-3,4,5,6-tetrahydro-3,8'-lign-8-ene-3,3',4,4',6-pentol; (3*R**,4*R**,7′S*,8′*R**)-form	-
40	4,7'-epoxy-3,4,5,6-tetrahydro-3,8'-lign-8-ene-3,3',4,4',6-pentol; (3*S*,4*R*,6S,7′*S*,8′*R*)-form	-
41	6'-hydroxypestalotiopsone C	*Acremonium strictum* [31]
42	dihydroepiheveadride	Unidentified fungus IFM 52672 [32]
43	4'-O-Acetylsinapyl angelate	*Solidago decurrens*
44	Nervolan A	*Inula nervosa* [33]
45	15-hydroxy-1(10),4,11(13)-germacratrien-12,6-olid-14-oic acid; (1(10,*E*,4*Z*,6α)-form, Me ester, Ac	*Oyedaea verbesinoides* [34]
46	2',3'-Isopropylideneobtusinin	*Pterocaulon alopecuroides* [35]
47	5-(Hydroxymethyl)-7-methyl-6-(2,4,5-trimethoxyphenyl)-8-oxabicyclo[3.2.1]oct-3-en-2-one	-
48	Acorusin C	*Acorus tatarinowii* [36]
49	Acortatarone A	*Acorus tatarinowii* [36]
50	5-(Hydroxymethyl)-7-methyl-6-(2,4,5-trimethoxyphenyl)-8-oxabicyclo[3.2.1]oct-3-en-2-one; (1*S*,5*S*,6*R*,7*S*)-form	-
51	3-Hydroxyviscidone	*Gerbera saxatilis* [37]
52	Ilexisochroman	*Ilex pubescens* [38]
53	2,7'-Lignan-3,3',4,4',5,5'-hexol	-
54	2,7'-Lignan-3,3',4,4',5,5'-hexol, *(R)*-form	-
55	3,8'-Lignan-3',4,4',5,9,9'-hexol	-
56	3,8'-Lignan-3',4,4',5,9,9'-hexol, *(R)*-form	-
57	3,8'-Lignan-3',4,4',5,9,9'-hexol, *(S)*-form	-
58	3,8'-Lignan-3',4,4',5,9,9'-hexol, *(ξ)*-form	-
59	8,8'-Lignan-2,2',4,4',5,5'-hexol	-
60	8,8'-Lignan-2,2',4,4',5,5'-hexol;(8*R**,8'*S**)-form	-
61	8,8'-Lignan-2,2',4,4',5,5'-hexol;(8*RS*,8'*SR*)-form	-
62	8,8'-Lignan-2,3',4,4',5,7'-hexol	-
63	8,8'-Lignan-2,3',4,4',5,7'-hexol;(7'*S*,8*S*,8'*R*)-form	-
64	8,8'-Lignan-2,3',4,4',5,7'-hexol;(8ξ,8'ξ)-form	-
65	8,8'-Lignan-3,3',4,4',5,5'-hexol	-
66	8,8'-Lignan-3,3',4,4',5,5'-hexol;(8*R*,8'*R*)-form	-
67	8,8'-Lignan-3,3',4,4',5,5'-hexol;(8*RS*,8'*SR*)-form	-
68	8,8'-Lignan-3,3',4,4',5,5'-hexol;(8ξ,8'ξ)-form	-
69	8,8'-Lignan-3,3',4,4',5,7-hexol	-
70	8,8'-Lignan-3,3',4,4',5,7-hexol;(7ξ,8ξ,8'ξ)-form	-

71	8,8'-Lignan-3,3',4,4',5,9'-hexol	-
72	8,8'-Lignan-3,3',4,4',5,9-hexol	-
73	8,8'-Lignan-3,3',4,4',5,9-hexol;(8*S*,8'*R*)-form	-
74	8,8'-Lignan-3,3',4,4',5,9′-hexol;(8*S*,8'*R*)-form	-
75	8,8'-Lignan-3,3',4,4',7,7'-hexol	-
76	8,8'-Lignan-3,3',4,4',7,7'-hexol;(7*RS*,7'*RS*,8*SR*,8'*SR*)-form	-
77	8,8'-Lignan-3,3',4,4',7,7'-hexol;(7*RS*,7'*SR*,8*RS*,8'*RS*)-form	-
78	8,8'-Lignan-3,3',4,4',7,7'-hexol;(7*S*,7'*S*,8*S*,8'*S*)-form	-
79	8,8'-Lignan-3,3',4,4',9,9'-hexol	-
80	8,8'-Lignan-3,3',4,4',9,9'-hexol;(8*R**,8'*R**)-form	-
81	8,8'-Lignan-3,3',4,4',9,9'-hexol;(8*R*,8'*R*)-form	-
82	8,8'-Lignan-3,3',4,4',9,9'-hexol;(8*R**,8'*S**)-form	-
83	8,8'-Lignan-3,3',4,4',9,9'-hexol;(8*R*S,8'*RS*)-form	-
84	8,8'-Lignan-3,3',4,4',9,9'-hexol;(8*R*S,8'*SR*)-form	-
85	8,8'-Lignan-3,3',4,4',9,9'-hexol;(8S,8'*S*)-form	-
86	8,8'-Lignan-3,3',4,4',9,9'-hexol;(8ξ,8'ξ)-form	-
87	8,8'-Lignan-3,3',5,5',7,7'-hexol	-
88	8,8'-Lignan-3,3',5,5',7,7'-hexol;(7*R**,7'*R**,8*R**,8'*S**)-form	-
89	2β-Hydroxynagilactone L	*Podocarpus nagi* [39]
90	3β-Hydroxynagilactone L	*Podocarpus nagi* [39]
91	15-Hydroxynagilactone L	*Podocarpus nagi* [39]
92	Laxiflorin U	*Isodon eriocalyx* var. *laxiflora* [40]
93	Andrographidoid D	*Andrographis paniculata* [41]
94	Octandrenolone;3,4-Dihydro, 3α,4β-dihydroxy	*Melicope erromangensis* [42]
95	Octandrenolone;9,10-Dihydro, 9α,10β-dihydroxy	*Melicope erromangensis* [42]
96	Oncocalyxone D;11-Me ether(11β-)	*Cordia oncocalyx* [43]
97	Oxirapentyne B	*Isaria felina* (DC.) Fr [44]
98	Oxirapentyne C	*Isaria felina* (DC.) Fr [44]
99	4,8'-Oxylignan-3,3',4',7',9-pentol	-
100	4,8'-Oxylignan-3,3',4',7',9-pentol;(7'*R*,8'*R*)-form	-
101	4,8'-Oxylignan-3,3',4',7',9-pentol;(7'*R**,8'*R**)-form	-
102	4,8'-Oxylignan-3,3',4',7',9-pentol;(7'*R*,8'S)-form	-
103	4,8'-Oxylignan-3,3',4',7',9-pentol;(7'*R*,8'S)-form	-
104	4,8'-Oxylignan-3,3',4',7',9-pentol;(7'*S*,8'S)-form	-
105	4,8'-Oxylignan-3,3',4',9,9'-pentol	-
106	4,8'-Oxylignan-3,3',4',9,9'-pentol;(*R*)-form	-
107	4,8'-Oxylignan-3,3',4',9,9'-pentol;(*S*)-form	-
108	4,8'-Oxylignan-3,4',7',9,9'-pentol	-
109	4,8'-Oxylignan-3,4',7',9,9'-pentol;(7′*R*S,8'*RS*)-form	-
110	4,8'-Oxylignan-3,4',7',9,9'-pentol;(7′*R*S,8'*SR*)-form	-
111	Strebluslignanol H	*Streblus asper* [45]
112	Streblusol D	*Streblus asper* [46]
113	tetrodecamycin	*Streptomyces nashvillensis* MJ885-mF8 [47]
114	1,6,10-trihydroxy-7(11),8-eremophiladien-12,8-olide;(1β,6β,10α)-form,1-Ketone, 6-propanoyl	*Senecio nemorensis*
115	8-Acryloylhydroxyisonobilin	*Anthemis nobilis* [48]
116	Combretastatin B1	*Combretum caffrum* [49] and *Combretum kraussi*

117	Bussealin B	*Bussea sakalava* [50]
118	11,12-Epoxyzearalenone	*Fusarium graminearum* [51]
119	4R-Hydroxyzearalenone	*Fusarium* sp. PSU-ES123 [52]
120	5R-Hydroxyzearalenone	*Gibberella zeae, Fusarium graminearium* and the marine-derived *Fusarium* sp. 05ABR26 [53]
121	5S-Hydroxyzearalenone	*Gibberella zeae* and *Fusarium graminearium* [51]
122	6R-Hydroxyzearalenone	*Fusarium* sp. PSU-ES123 [52]
123	8R-Hydroxyzearalenone	*Fusarium* sp. PSU-ES73 [54]
124	8S-Hydroxyzearalenone	*Fusarium* sp. PSU-ES123 [52]
125	10-Hydroxyzearalenone	*Fusarium graminearum* [51]

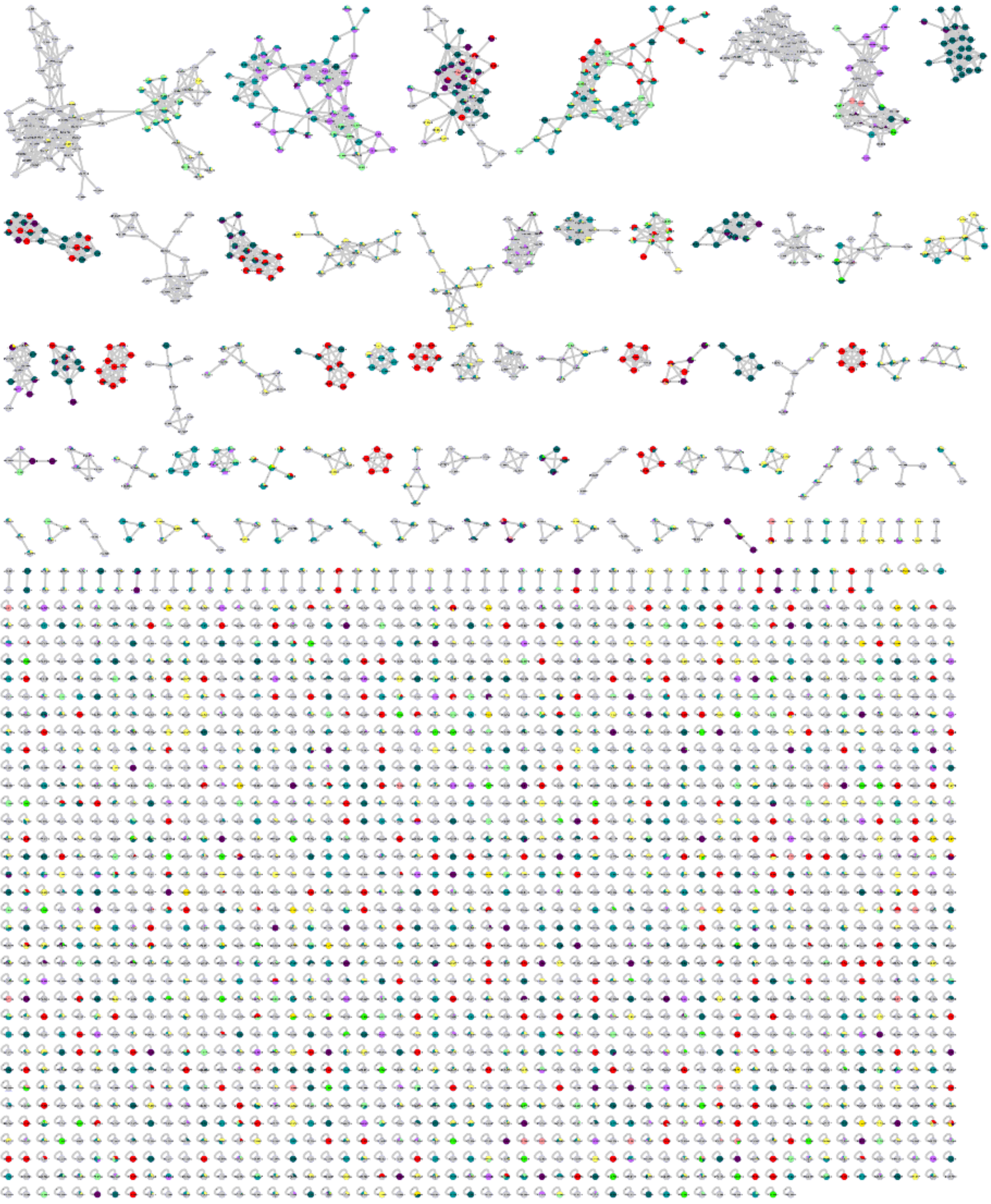

Figure S1. The molecular networking of the strain extracts from different condition cultures and the control extracts. Different culture condition is represented by different color code.

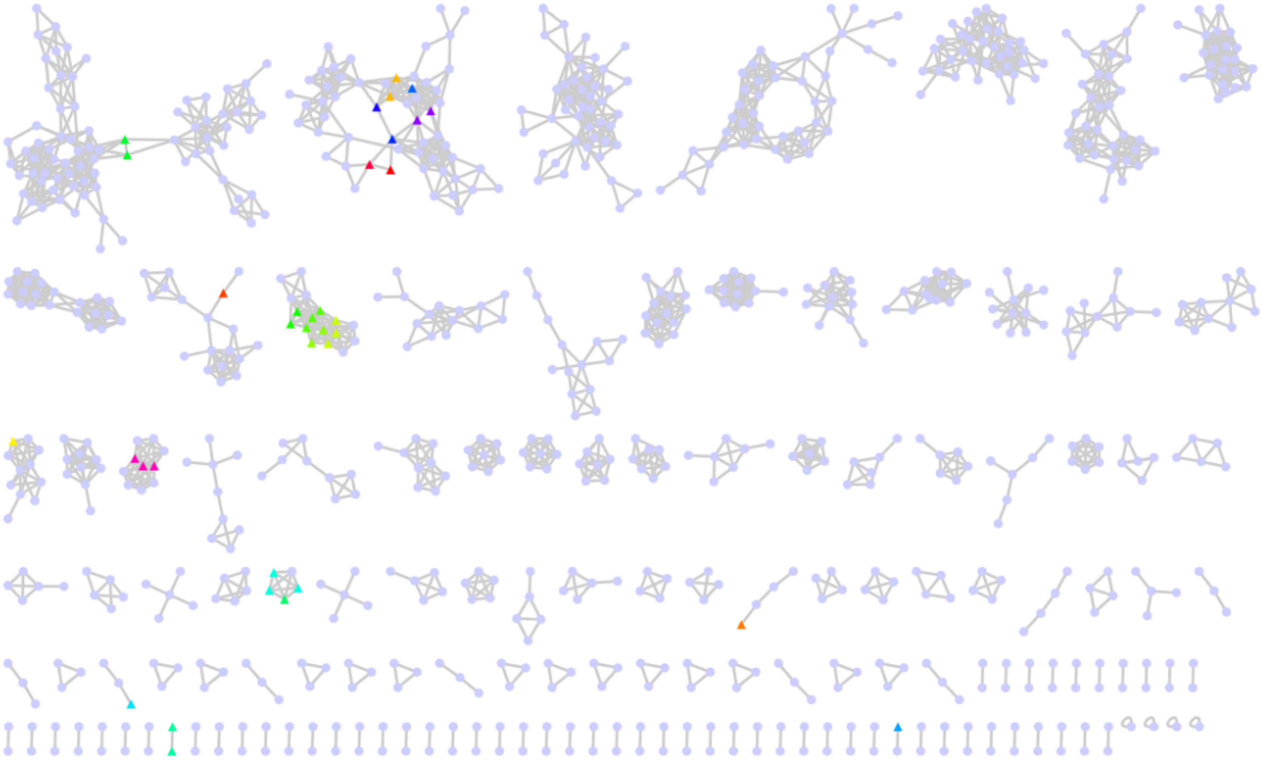

Figure S2. The molecular networking with dereplicated nodes that are mapped by rainbow color codes while others stayed purple.

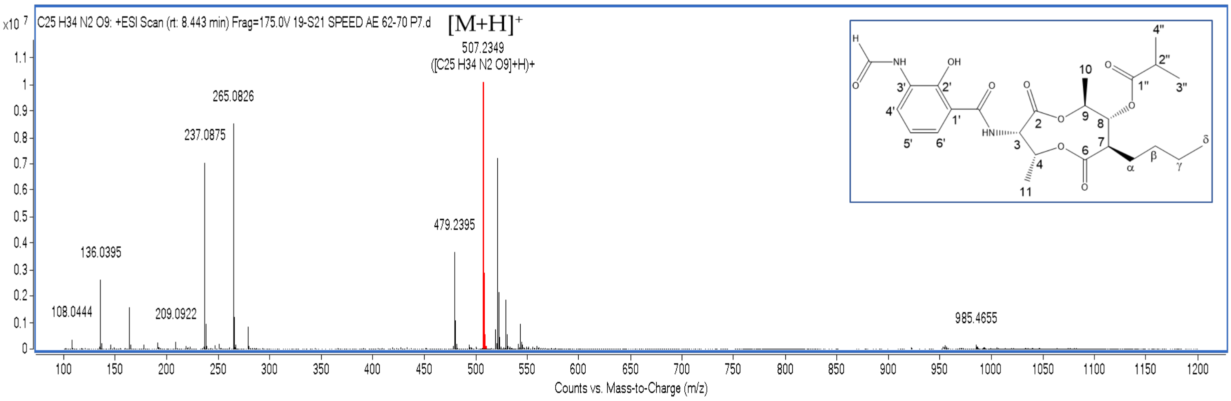

Figure S3. The HRESIMS spectrum of Antimycin A4a

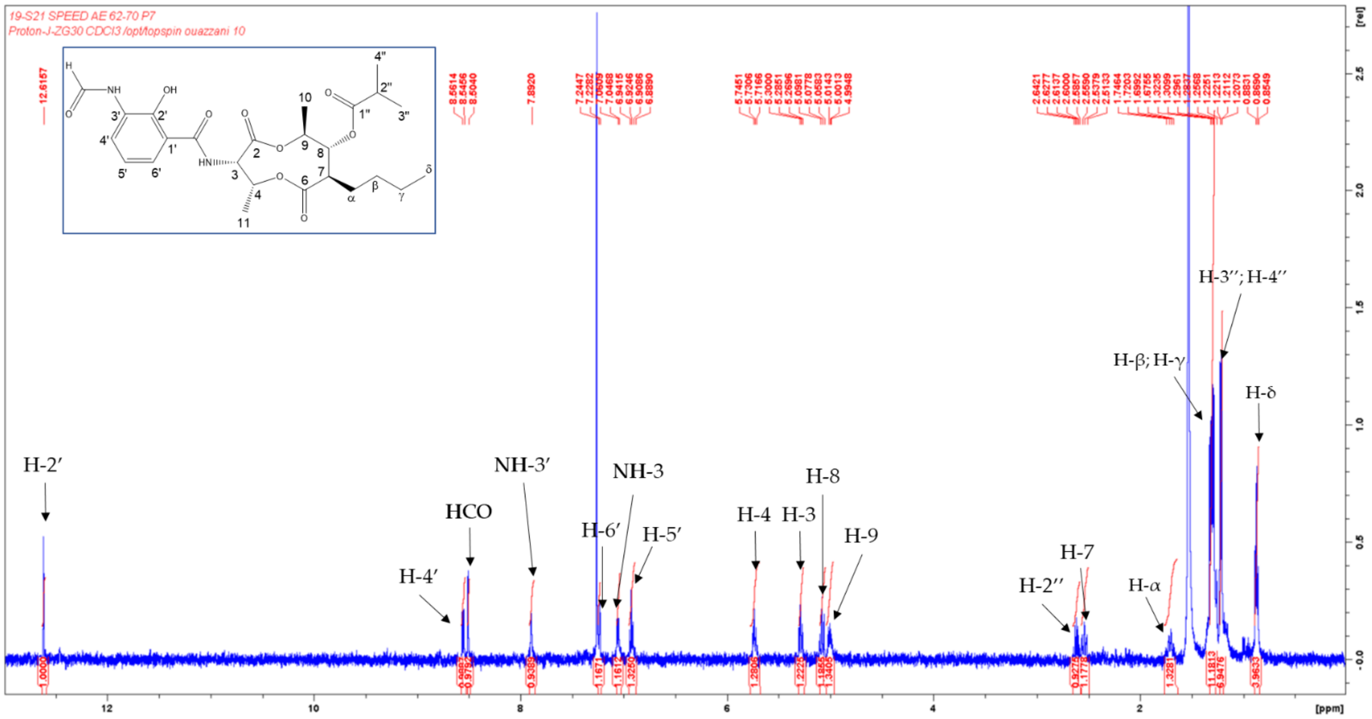

Figure S4. ^{1}H NMR spectrum of Antimycin A4a in $CDCl_3$ (500 MHz)

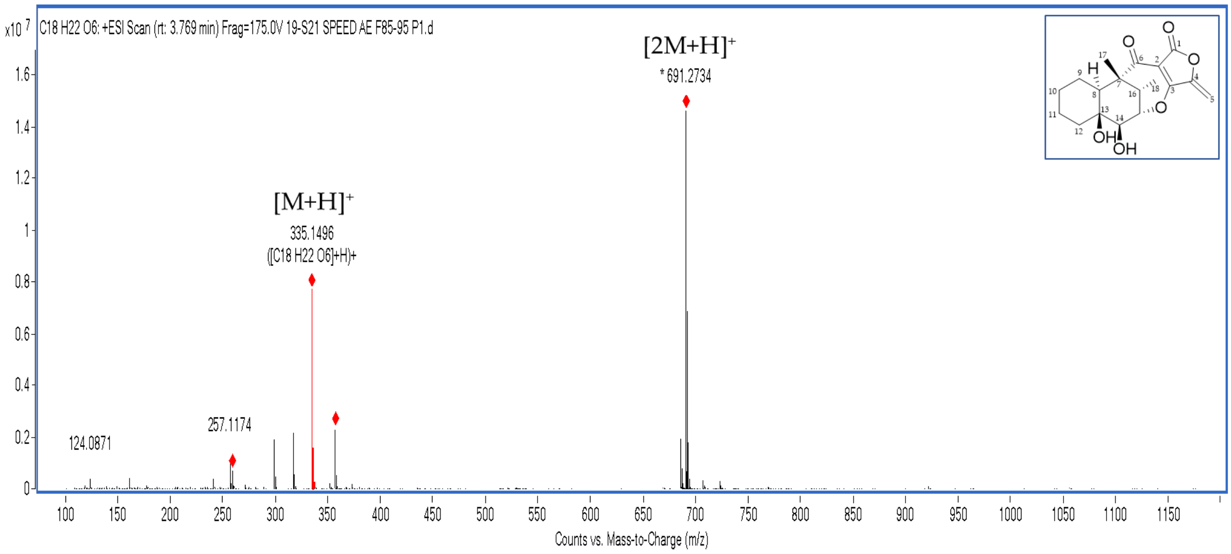

Figure S5. The HRESIMS spectrum of tetrodecamycin

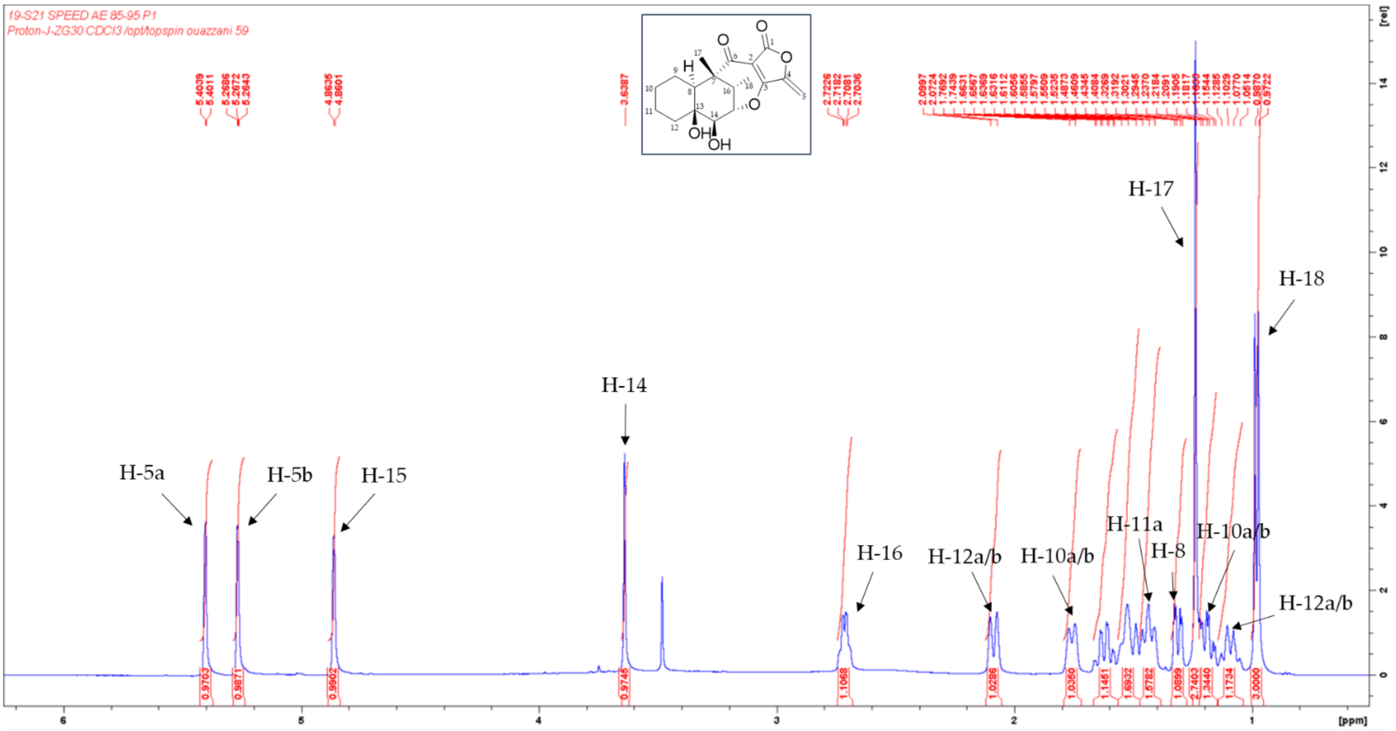

Figure S6. ^{1}H NMR spectrum of tetrodecamycin in $CDCl_3$ (500 MHz)

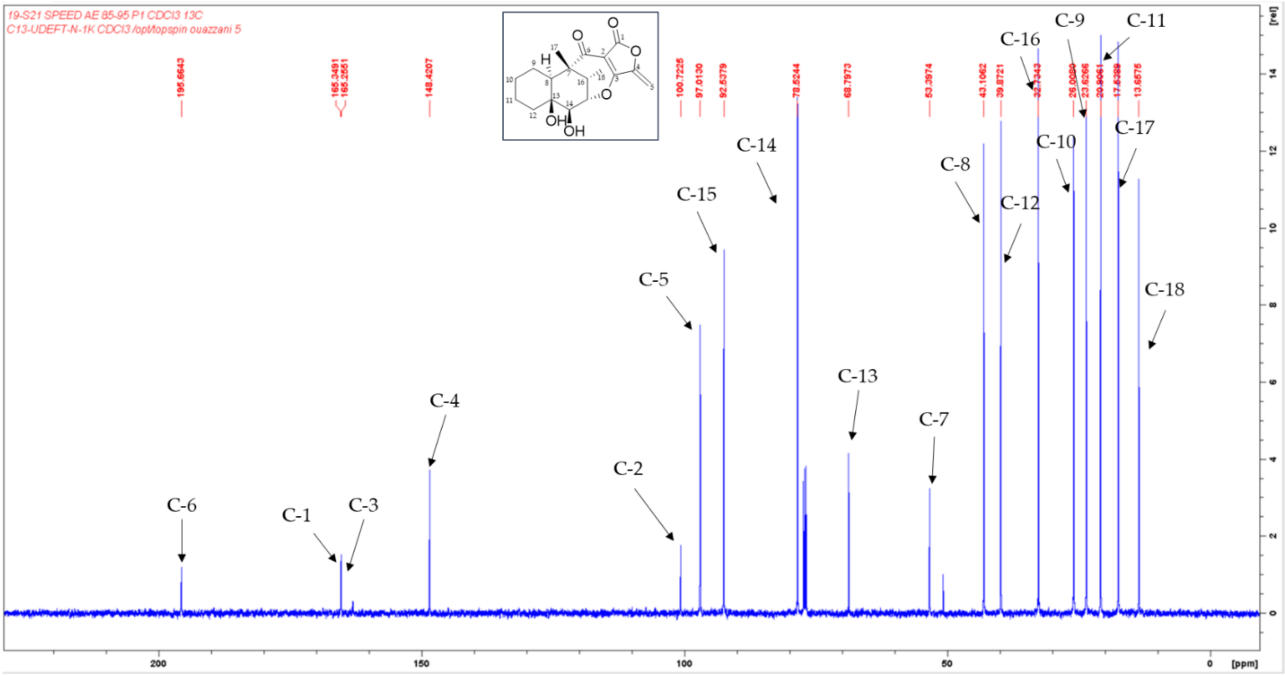

Figure S7. ^{13}C NMR spectrum of tetrodecamycin in $CDCl_3$ (125 MHz)

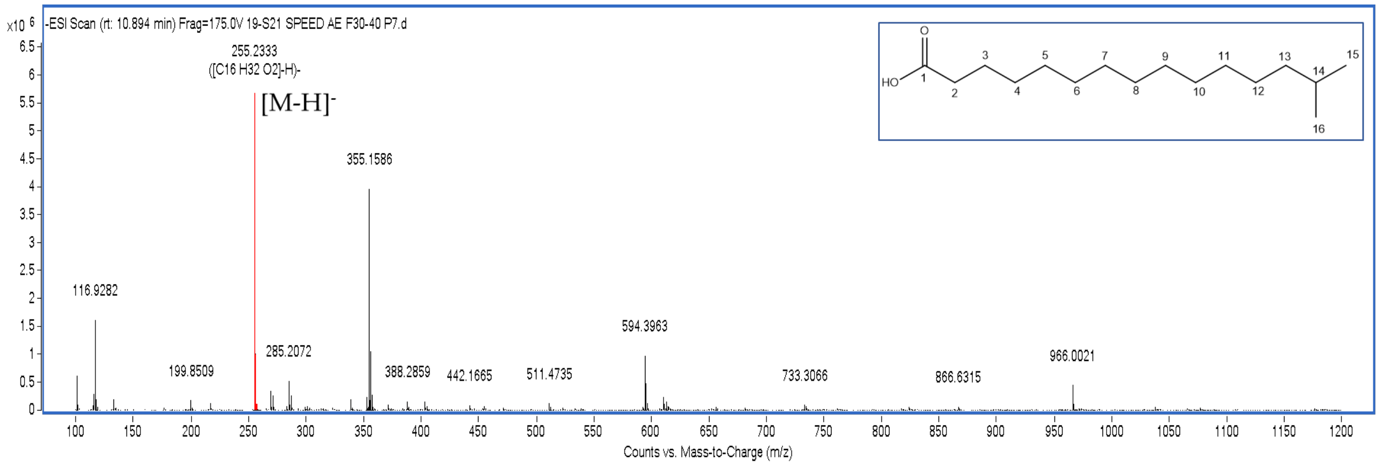

Figure S8. The HRESIMS spectrum of 14-methylpentadecanoic acid

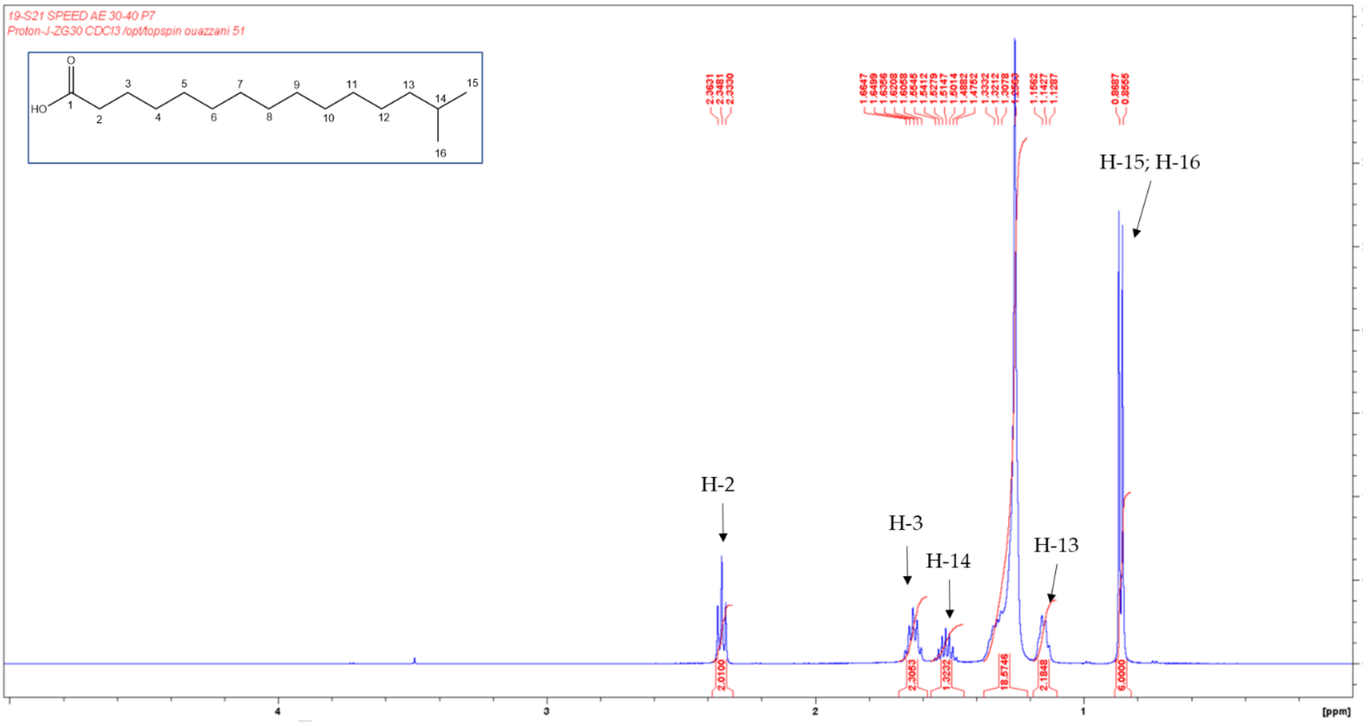

Figure S9. ^{1}H NMR spectrum of 14-methylpentadecanoic acid in $CDCl_3$ (500 MHz)

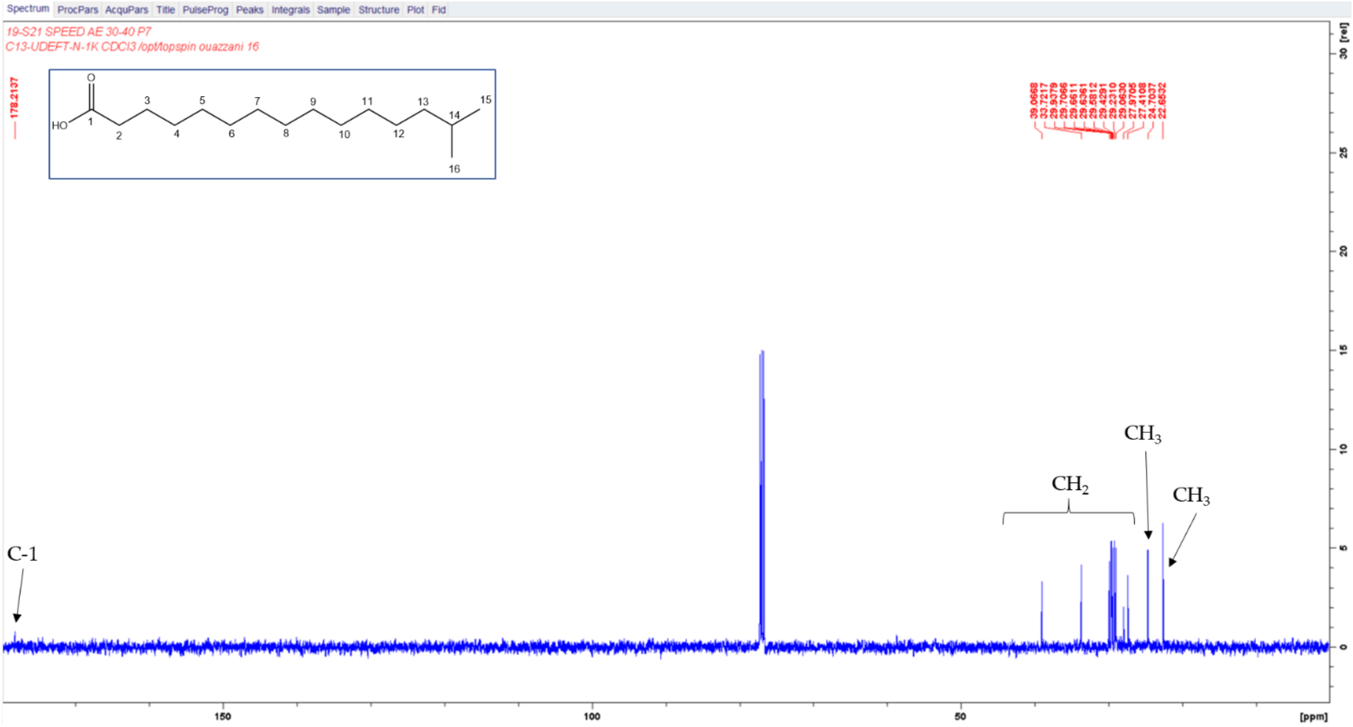

Figure S10. ^{13}C NMR spectrum of 14-methylpentadecanoic acid in $CDCl_3$ (125 MHz)

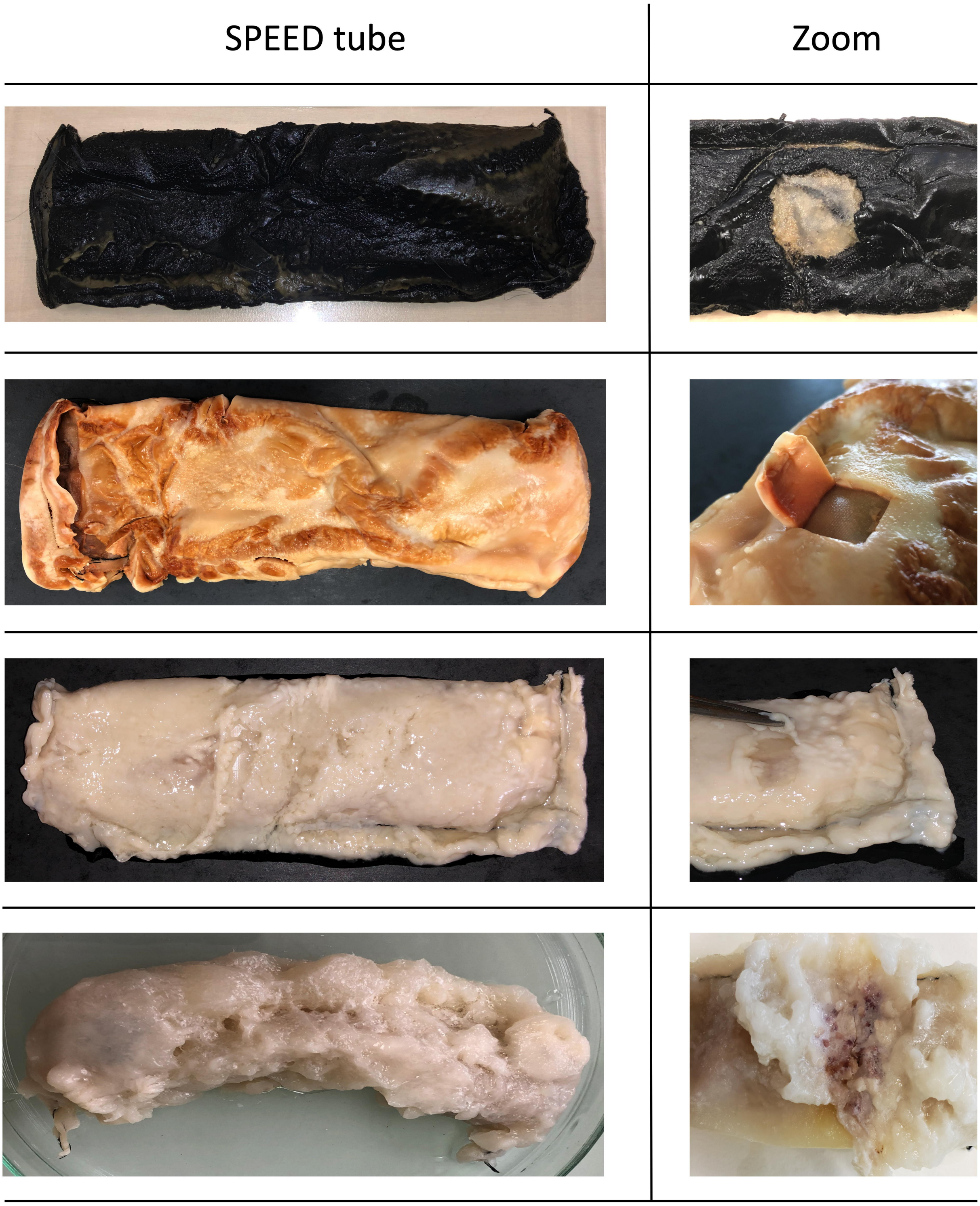

Figure S11. Further SPEED pictures

References

1. Takada, K.; Ninomiya, A.; Naruse, M.; Sun, Y.; Miyazaki, M.; Nogi, Y.; Okada, S.; Matsunaga, S., Surugamides A-E, cyclic octapeptides with four D-amino acid residues, from a marine Streptomyces sp.: LC-MS-aided inspection of partial hydrolysates for the distinction of D- and L-amino acid residues in the sequence. *The Journal of organic chemistry* **2013,** *78* (13), 6746-50.
2. Yamanaka, K.; Oikawa, H.; Ogawa, H. O.; Hosono, K.; Shinmachi, F.; Takano, H.; Sakuda, S.; Beppu, T.; Ueda, K., Desferrioxamine E produced by Streptomyces griseus stimulates growth and development of Streptomyces tanashiensis. *Microbiology* **2005,** *151* (Pt 9), 2899-2905.
3. Martinez, J. S.; Haygood, M. G.; Butler, A., Identification of a natural desferrioxamine siderophore produced by a marine bacterium. *Limnology and Oceanography* **2001,** *46* (2), 420-424.
4. Shaaban, K. A.; Singh, S.; Elshahawi, S. I.; Wang, X.; Ponomareva, L. V.; Sunkara, M.; Copley, G. C.; Hower, J. C.; Morris, A. J.; Kharel, M. K.; Thorson, J. S., Venturicidin C, a new 20-membered macrolide produced by Streptomyces sp. TS-2-2. *J Antibiot (Tokyo)* **2014,** *67* (3), 223-30.
5. Lee, H.-S.; Shin, H. J.; Jang, K. H.; Kim, T. S.; Oh, K.-B.; Shin, J., Cyclic Peptides of the Nocardamine Class from a Marine-Derived Bacterium of the Genus Streptomyces. *Journal of Natural Products* **2005,** *68* (4), 623-625.
6. Mortazavi, M.; Akbarzadeh, A., Improvement of Desferrioxamine B Production of Streptomyces pilosus ATCC 19797 With Use of Protease Inhibitor and Minerals Related to Its Activity. *Indian journal of clinical biochemistry : IJCB* **2012,** *27* (3), 274-7.
7. Barrow, C. J.; Oleynek, J. J.; Marinelli, V.; Sun, H. H.; Kaplita, P.; Sedlock, D. M.; Gillum, A. M.; Chadwick, C. C.; Cooper, R., Antimycins, inhibitors of ATP-citrate lyase, from a Streptomyces sp. *J Antibiot (Tokyo)* **1997,** *50* (9), 729-33.
8. Datta, S. C.; Ghosh, J. J., Effect of citreoviridin, a toxin from Penicillium citreoviride NRRL 2579, on glycogen metabolism of rat brain. *Toxicon* **1981,** *19* (2), 217-222.
9. Wronska, A. K.; Bogus, M. I., Harman and norharman, metabolites of the entomopathogenic fungus Conidiobolus coronatus (Entomophthorales), affect the serotonin levels and phagocytic activity of hemocytes, insect immunocompetent cells, in Galleria mellonella (Lepidoptera). *Cell & bioscience* **2019,** *9*, 29.
10. Wattana-Amorn, P.; Charoenwongsa, W.; Williams, C.; Crump, M. P.; Apichaisataienchote, B., Antibacterial activity of cyclo(L-Pro-L-Tyr) and cyclo(D-Pro-L-Tyr) from Streptomyces sp. strain 22-4 against phytopathogenic bacteria. *Natural product research* **2016,** *30* (17), 1980-1983.
11. Isaka, M.; Suyarnsestakorn, C.; Tanticharoen, M.; Kongsaeree, P.; Thebtaranonth, Y., Aigialomycins A−E, New Resorcylic Macrolides from the Marine Mangrove Fungus Aigialus parvus. *The Journal of organic chemistry* **2002,** *67* (5), 1561-1566.
12. Wu, Z.; Lai, Y.; Zhou, L.; Wu, Y.; Zhu, H.; Hu, Z.; Yang, J.; Zhang, J.; Wang, J.; Luo, Z.; Xue, Y.; Zhang, Y., Enantiomeric Lignans and Neolignans from Phyllanthus glaucus: Enantioseparation and Their Absolute Configurations. *Scientific reports* **2016,** *6*, 24809.
13. Majumder, P. L.; Pal, S., Cumulatin and tristin, two bibenzyl derivatives from the orchids Dendrobium cumulatum and Bulbophyllum triste. *Phytochemistry* **1993,** *32* (6), 1561-1565.
14. Szwalbe, A. J.; Williams, K.; O'Flynn, D. E.; Bailey, A. M.; Mulholland, N. P.; Vincent, J. L.; Willis, C. L.; Cox, R. J.; Simpson, T. J., Novel nonadride, heptadride and maleic acid metabolites from the byssochlamic acid producer Byssochlamys fulva IMI 40021 - an insight into the biosynthesis of maleidrides. *Chemical communications* **2015,** *51* (96), 17088-91.
15. Li, L. M.; Zou, Q.; Li, G. Y., Chromones from an ascomycete, Chaetomium aureus. *Chinese Chemical Letters* **2010,** *21* (10), 1203-1205.
16. Huang, H.; Feng, X.; Xiao, Z. e.; Liu, L.; Li, H.; Ma, L.; Lu, Y.; Ju, J.; She, Z.; Lin, Y., Azaphilones and p-Terphenyls from the Mangrove Endophytic Fungus Penicillium chermesinum (ZH4-E2) Isolated from the South China Sea. *Journal of Natural Products* **2011,** *74* (5), 997-1002.
17. Pettit, G. R.; Cragg, G. M.; Herald, D. L.; Schmidt, J. M.; Lohavanijaya, P., Isolation and structure of combretastatin. *Canadian Journal of Chemistry* **1982,** *60* (11), 1374-1376.
18. Achenbach, H.; Mühlenfeld, A.; Brillinger, G. U., Stoffwechselprodukte von Mikroorganismen, XXX. Phthalide und Chromanole aus Aspergillus duricaulis. *Liebigs Annalen der Chemie* **1985,** *1985* (8), 1596-1628.
19. Paranagama, P. A.; Wijeratne, E. M. K.; Gunatilaka, A. A. L., Uncovering Biosynthetic Potential of Plant-Associated Fungi: Effect of Culture Conditions on Metabolite Production by Paraphaeosphaeria quadriseptata and Chaetomium chiversii. *Journal of Natural Products* **2007,** *70* (12), 1939-1945.
20. Gao, J.; Radwan, M. M.; Leon, F.; Dale, O. R.; Husni, A. S.; Wu, Y.; Lupien, S.; Wang, X.; Manly, S. P.; Hill, R. A.; Dugan, F. M.; Cutler, H. G.; Cutler, S. J., Neocosmospora sp.-derived resorcylic acid lactones with in vitro binding affinity for human opioid and cannabinoid receptors. *J Nat Prod* **2013,** *76* (5), 824-8.
21. Ayer, W. A.; Lee, S. P.; Tsuneda, A.; Hiratsuka, Y., The isolation, identification, and bioassay of the antifungal metabolites produced by Monocillium nordinii. *Canadian Journal of Microbiology* **1980,** *26* (7), 766-773.

22. Xu, L.; Wu, P.; Xue, J.; Molnar, I.; Wei, X., Antifungal and Cytotoxic beta-Resorcylic Acid Lactones from a Paecilomyces Species. *J Nat Prod* **2017,** *80* (8), 2215-2223.
23. Wang, X. R.; Wu, Q. X.; Shi, Y. P., Terpenoids and sterols from Saussurea cauloptera. *Chemistry & biodiversity* **2008,** *5* (2), 279-89.
24. Wu, W.; Bi, X.-L.; Cao, J.-Q.; Zhang, K.-Q.; Zhao, Y.-Q., New antitumor compounds from Carya cathayensis. *Bioorganic & Medicinal Chemistry Letters* **2012,** *22* (5), 1895-1898.
25. Ito, M.; Matsuzaki, K.; Wang, J.; Daikonya, A.; Wang, N. L.; Yao, X. S.; Kitanaka, S., New phenanthrenes and stilbenes from Dendrobium loddigesii. *Chemical & pharmaceutical bulletin* **2010,** *58* (5), 628-33.
26. Zhang, X.; Gao, H.; Wang, N.-L.; Yao, X.-S., Three new bibenzyl derivatives from Dendrobium nobile. *Journal of Asian Natural Products Research* **2006,** *8* (1-2), 113-118.
27. Wang, Y. X.; Ren, Q.; Yan, Z. Y.; Wang, W.; Zhao, L.; Bai, M.; Wang, X. B.; Huang, X. X.; Song, S. J., Flavonoids and their derivatives with beta-amyloid aggregation inhibitory activity from the leaves and twigs of Pithecellobium clypearia Benth. *Bioorg Med Chem Lett* **2017,** *27* (21), 4823-4827.
28. Wang, L. Q.; Wu, M. M.; Liu, J. P.; Li, Y.; Hua, Y.; Wang, Y. Y.; Li, X. Y.; Chen, Y. G.; Wang, J. H., Five new diarylpropan-1-ols from Combretum yunnanense. *Planta medica* **2011,** *77* (16), 1841-4.
29. Seca, A. M. L.; Silva, A. M. S.; Silvestre, A. J. D.; Cavaleiro, J. A. S.; Domingues, F. M. J.; Pascoal-Neto, C., Lignanamides and other phenolic constituents from the bark of kenaf (Hibiscus cannabinus). *Phytochemistry* **2001,** *58* (8), 1219-1223.
30. Bohlmann, F.; Singh, P.; Jakupovic, J.; Robinson, H.; King, R. M., An epoxygermacranolide and further constituents from Mikania species. *Phytochemistry* **1982,** *21* (3), 705-707.
31. Hammerschmidt, L.; Debbab, A.; Ngoc, T. D.; Wray, V.; Hemphil, C. P.; Lin, W.; Broetz-Oesterhelt, H.; Kassack, M. U.; Proksch, P.; Aly, A. H., Polyketides from the mangrove-derived endophytic fungus Acremonium strictum. *Tetrahedron Letters* **2014,** *55* (24), 3463-3468.
32. Hosoe, T.; Fukushima, K.; Itabashi, T.; Nozawa, K.; Takizawa, K.; Okada, K.; Takaki, G. M.; Kawai, K., A new nonadride derivative, dihydroepiheveadride, as characteristic antifungal agent against filamentous fungi, isolated from unidentified fungus IFM 52672. *J Antibiot (Tokyo)* **2004,** *57* (9), 573-8.
33. Yan, L.; Huang, Y.; Fu, J.-J.; Qin, J.-J.; Zeng, Q.; Zhu, Y.; Yan, S. K.; Zhang, W.-D.; Jin, H.-Z., Three New Phenylpropanoids from Inula nervosa Wall. *Helvetica Chimica Acta* **2010,** *93* (7), 1418-1421.
34. Stokes, S.; Castro, V.; Poveda, L.; Papastergiou, F.; Jakupovic, J., Melampolides from Oyedaea verbesinoides. *Phytochemistry* **1992,** *31* (8), 2894-2896.
35. Alarcón, R.; Pacciaroni, A.; Peñaloza, L.; Uriburu, M. L.; Boemo, A.; Sosa, V., Phenolic compounds from Pterocaulon alopecuroides. *Biochemical Systematics and Ecology* **2010,** *38* (5), 1059-1064.
36. Ni, G.; Shi, G. R.; Zhang, D.; Fu, N. J.; Yang, H. Z.; Chen, X. G.; Yu, D. Q., Cytotoxic Lignans and Sesquiterpenoids from the Rhizomes of Acorus tatarinowii. *Planta medica* **2016,** *82* (7), 632-8.
37. Chen, Y.-J.; Li, Y.; Chen, J.-J.; Gao, K., Benzofuran Derivatives from Gerbera saxatilis. *Helvetica Chimica Acta* **2007,** *90* (1), 176-182.
38. Lin, L.-P.; Kong, X.; Chen, L.; Chen, L., Chemical constituents from the roots of cultivated Ilex pubescens. *Biochemical Systematics and Ecology* **2019,** *82*, 13-15.
39. Feng, Z. L.; Zhang, L. L.; Zheng, Y. D.; Liu, Q. Y.; Liu, J. X.; Feng, L.; Huang, L.; Zhang, Q. W.; Lu, J. J.; Lin, L. G., Norditerpenoids and Dinorditerpenoids from the Seeds of Podocarpus nagi as Cytotoxic Agents and Autophagy Inducers. *J Nat Prod* **2017,** *80* (7), 2110-2117.
40. Wang, W.-G.; Du, X.; Li, X.-N.; Yan, B.-C.; Zhou, M.; Wu, H.-Y.; Zhan, R.; Dong, K.; Pu, J.-X.; Sun, H.-D., Four new diterpenoids from Isodon eriocalyx var. laxiflora. *Natural products and bioprospecting* **2013,** *3* (4), 145-149.
41. Xu, C.; Chou, G. X.; Wang, C. H.; Wang, Z. T., Rare noriridoids from the roots of Andrographis paniculata. *Phytochemistry* **2012,** *77*, 275-9.
42. Muyard, F.; Bissoue, A. N.; Bevalot, F.; Tillequin, F.; Cabalion, P.; Vaquette, J., Acetophenones and other constituents from the roots of Melicope erromangensis. *Phytochemistry* **1996,** *42* (4), 1175-1179.
43. Matos, T. S.; Silva, A. K. O.; Quintela, A. L.; Francisco das Chagas Pinto, L.; Canuto, K. M.; Braz-Filho, R.; Fonseca, M. J. S.; Luna-Costa, A. M.; Paz, I. A.; Nascimento, N. R. F.; Silveira, E. R.; Pessoa, O. D. L., Neuroinhibitory meroterpenoid compounds from Cordia oncocalyx. *Fitoterapia* **2017,** *123*, 65-72.
44. Smetanina, O. F.; Yurchenko, A. N.; Afiyatullov, S. S.; Kalinovsky, A. I.; Pushilin, M. A.; Khudyakova, Y. V.; Slinkina, N. N.; Ermakova, S. P.; Yurchenko, E. A., Oxirapentyns B–D produced by a marine sediment-derived fungus Isaria felina (DC.) Fr. *Phytochemistry Letters* **2012,** *5* (1), 165-169.
45. Chen, H.; Li, J.; Wu, Q.; Niu, X. T.; Tang, M. T.; Guan, X. L.; Li, J.; Yang, R. Y.; Deng, S. P.; Su, X. J., Anti-HBV activities of Streblus asper and constituents of its roots. *Fitoterapia* **2012,** *83* (4), 643-9.
46. Li, J.; Huang, Y.; Guan, X. L.; Li, J.; Deng, S. P.; Wu, Q.; Zhang, Y. J.; Su, X. J.; Yang, R. Y., Anti-hepatitis B virus constituents from the stem bark of Streblus asper. *Phytochemistry* **2012,** *82*, 100-9.

47. Tsuchida, T.; Iinuma, H.; Nishida, C.; Kinoshita, N.; Sawa, T.; Hamada, M.; Takeuchi, T., Tetrodecamycin and dihydrotetrodecamycin, new antimicrobial antibiotics against Pasteurella piscicida produced by Streptomyces nashvillensis MJ885-mF8. I. Taxonomy, fermentation, isolation, characterization and biological activities. *J Antibiot (Tokyo)* **1995,** *48* (10), 1104-9.
48. De Mieri, M.; Monteleone, G.; Ismajili, I.; Kaiser, M.; Hamburger, M., Antiprotozoal Activity-Based Profiling of a Dichloromethane Extract from Anthemis nobilis Flowers. *J Nat Prod* **2017,** *80* (2), 459-470.
49. Pettit, G. R.; Singh, S. B.; Niven, M. L.; Hamel, E.; Schmidt, J. M., Isolation, Structure, and Synthesis of Combretastatins A-1 and B-1, Potent New Inhibitors of Microtubule Assembly, Derived from Combretum caffrum. *Journal of Natural Products* **1987,** *50* (1), 119-131.
50. Pan, E.; Harinantenaina, L.; Brodie, P. J.; Miller, J. S.; Callmander, M. W.; Rakotonandrasana, S.; Rakotobe, E.; Rasamison, V. E.; Kingston, D. G. I., Four Diphenylpropanes and a Cycloheptadibenzofuran from Bussea sakalava from the Madagascar Dry Forest. *Journal of Natural Products* **2010,** *73* (11), 1792-1795.
51. Shen, W.; Mao, H.; Huang, Q.; Dong, J., Benzenediol lactones: a class of fungal metabolites with diverse structural features and biological activities. *European journal of medicinal chemistry* **2015,** *97*, 747-77.
52. Saetang, P.; Rukachaisirikul, V.; Phongpaichit, S.; Sakayaroj, J.; Shi, X.; Chen, J.; Shen, X., β-Resorcylic macrolide and octahydronaphthalene derivatives from a seagrass-derived fungus Fusarium sp. PSU-ES123. *Tetrahedron* **2016,** *72* (41), 6421-6427.
53. Zhao, L. L.; Gai, Y.; Kobayashi, H.; Hu, C. Q.; Zhang, H. P., 5′-Hydroxyzearalenol, a new β-resorcylic macrolide from Fusarium sp. 05ABR26. *Chinese Chemical Letters* **2008,** *19* (9), 1089-1092.
54. Supaphon, P.; Arunpanichlert; Rukachaisirikul; Sukpondma, Y.; Phongpaichit; Supaphon; Sakayaroj, O., *A β-resorcylic macrolide from the seagrass-derived fungus Fusarium sp. PSU-ES73, Arch. Pharm. Res*. 2016.
